# The relationship between psychological contract and occupational wellbeing of mother–infant care helpers in Zhejiang Province

**DOI:** 10.1186/s12960-023-00793-w

**Published:** 2023-03-01

**Authors:** Qingge Li, Yacen Li, Ying Jin, Suwen Feng

**Affiliations:** Women’s Hospital School of Medicine Zhejiang University, No.1 Xue Shi Road, Hangzhou, 310006 Zhejiang Province China

**Keywords:** Psychological contract, Occupational wellbeing, Mother–infant care helpers

## Abstract

**Background:**

Mother–infant care (MIC) helpers have become an indispensable part in hospital services. In order to stabilize the MIC workforce, it is essential for administrators to have a solid understanding of what may influence occupational wellbeing. This article aims to explore how demographic characteristics and psychological contract affect occupational wellbeing among MIC helpers in Zhejiang Province, China.

**Methods:**

This is a quantitative, cross-sectional study with MIC helpers in obstetrics from 20 hospitals in Zhejiang Province. A questionnaire including demographic data, a psychological contract scale and an occupational wellbeing scale was used in this study. Multiple linear regression was conducted to investigate the relationships between demographic characteristics, psychological contract and occupational wellbeing.

**Results:**

This study surveyed 260 MIC helpers and found out the mean score of the psychological contract was 4.38 and the mean score of the occupational wellbeing was 4.01. Monthly income and psychological contract were significant predictors of occupational wellbeing (*F* = 142.167, *p* < 0.001), which explained 62.1% of the total amount of variance in occupational wellbeing. Psychological contract was the most important predictor of occupational wellbeing.

**Conclusions:**

Administrators should pay attention to the effect of psychological contract on occupational wellbeing of the MIC helpers in China. Focusing on the inner needs should be considered as a strategy for stabilizing the team.

**Supplementary Information:**

The online version contains supplementary material available at 10.1186/s12960-023-00793-w.

## Introduction

In China, with the introduction of “Baby-friendly Initiative” in 1992, the mode of obstetric care was transformed from the form of mother–infant separation, centralized management of newborns and artificial feeding to mother–infant room care and breastfeeding encouragement [1, 2], which means the nurses’ job has changed from attending to the pregnant and parturient women to mother–infant care guidance and health education for the family with the puerperae and the newborn at the center. Moreover, during Covid 19 pandemic, in order to minimize the flow of people, only one fixed accompanying relative (usually the husband) is allowed in the obstetric ward. Inexperienced in breast feeding and newborn caring, these new parents are often overreacted to the newborn’s normal cry [3], which requires frequent visits by nurses to provide reassurance and emotional support. At the same time, needs arose for additional hospital beds to accommodate women with high-risk pregnancies and obstetric complications also led to an increasing workload [4]. According to the Statistical Bulletin of China’s Health Development in 2020, the coverage rate of nurses and/or midwives per 1000 population in China is merely 3.34 [5]. The available allocation of nursing human resources can not meet the increasing demands on a wide spectrum of care. The MIC helpers as a kind of nursing assistants become an integral part of in-hospital services to alleviate this situation.

The MIC helpers are those who provide care services for the pregnant, the parturient, and the infant in their daily lives [6], in a role similar to that of maternity support workers in the UK [7] or Aboriginal Maternal Infant Care workers in in Australia [8]. Mainly engaged in auxiliary nursing, they are not professional healthcare workers in medical institutions [9]. The MIC helper was developed from the Chinese term "Yuesao" (who takes care of postpartum women doing the month) [10]. Their workplaces can be hospitals, mother–infant service agencies (postpartum care centers), and the homes of the puerperae. The MIC helpers discussed in this article are mainly responsible for the care of the puerperae in the hospital within 2–5 days after parturition, and their job covers basic life care for puerperae, daily care for the newborns, etc. They are hired by the puerperae voluntarily according to their needs. The MIC helpers in the hospitals are usually trained and dispatched by the mother–infant service agencies and managed by both after the hospitals sign agreement with the agencies. The advent of this occupation marks a refined division of labor and individualization of social demands [11]. Partnership between the MIC helpers and obstetric nurses, enables the combination of holistic care and fragmented care to provide better services for maternal and infants’ health. Researches in recent years have shown that professional care of MIC helpers was beneficial to postpartum rehabilitation of puerpera, improve breastfeeding, hospital satisfaction, the ability of families to care for newborns, and reduce the incidence of breast engorgement [12, 13]. In 2016, with the implementation of China's two-children policy, the booming market brought about a huge demand for MIC helpers. In 2018, the Ministry of Human Resources and the Ministry of Social Security included MIC helpers as "urgently needed occupations" in the professional skill level recognition talent evaluation [14]. The number of MIC institutions has increased from more than 6000 in 2018 to more than 7300 in 2019, with an annual growth rate of 30% [15].

Currently, there is no regulation of this job role of MIC helpers, and the MIC helpers have many deficiencies in human resource management, system construction, and service provision [16], which seriously affect the stability of the MIC helpers and the improvement of service quality. There are few studies on MIC helpers in China, and even fewer high-quality articles. Occupational wellbeing is an important factor in employee’s decision to resign [17] and has an impact on the job performance [18], the interpersonal relationships [19]. As such, improving MIC helpers’ occupational wellbeing has become a major concern among administrators.

High levels of work-related wellbeing can be conceptualized as occupational wellbeing. The definition of occupational wellbeing varies within different fields of research. The International Labour Organization [20] considers occupational wellbeing as being constructed from all aspects of working life, including the workers’ perceptions of their work, the physical and mental working environment and the working community; considering these aspects will generate healthy, satisfied and engaged workers. The wellbeing was much studied in occupations such as physicians [19], nurses [18] and educators [21], and studies have shown that occupational wellbeing was influenced by leadership style, work environments, social support, employees' opportunities and work relationships [18, 22, 23]. Previous studies have mostly focused on the impacts of work-related factors. By contrast, there is a dearth of studies on personal factors. When the work-related factors cannot be changed, the personal factors become an effective strategy to improve occupational wellbeing. Thus, this article, by focusing on the inner needs of the MIC helpers, will explore the personal influencing factors of their occupational wellbeing.

In recent years, the research on the relationship between psychological contract and work-related outcomes is a growing concern [24, 25]. The psychological contract refers to the individual employee’s perception and belief system of the responsibilities and obligations between employees and employers, and it is about psychological expectations and commitment in the subjective sense [26]. Based on the Chinese cultural background, Chinese scholar Li Yuan [27] believes obligations in interpersonal communication and interpersonal environment are of great importance for Chinese employees. Therefore, he has put forward with three-dimension psychological contract, i.e., normal obligations, interpersonal obligations, and developmental obligations. Normal obligations emphasize on the explicit, concrete, and fundamental mutual obligations of employees and employers. Interpersonal obligations focus on the two parties’ social connections and mutual trust and respect. Developmental obligations highlight that both employers and employees take responsibility in career success and career development of each other. An Australian scholar [28] used psychological contract theory to explore its relationship with several attitudinal and mental health outcomes among registered nurses and midwives. An Irish scholar [25] found that there was a positive relationship between psychological contract and job satisfaction in doctors. In China, some scholars have already surveyed the psychological contract of medical care workers [16] and care helpers in elderly care institutions [29, 30].

Some demographic data have been found to be correlated with occupational wellbeing, and they should be taken into consideration when investigating the relationship between psychological contract and occupational wellbeing. Length of working, educational background, monthly income, grade of hospital, age and working hours [18, 31, 32] were found correlated with occupational wellbeing.

This study has two aims. First, to provide a profile of occupational wellbeing and psychological contract among MIC helpers in China. Second, to determine to what extent the variance in overall occupational wellbeing is explained by demographic data and psychological contract, so as provide management suggestions and countermeasures for improving the occupational wellbeing and stabilizing the team.

## Methods

### Sample and procedure

Convenience sampling was adopted to make a cross-sectional survey in 20 hospitals which employ MIC helpers in 9 cities of Zhejiang Province in January and February, 2022. The trained researchers and administrators of the MIC helpers in hospitals jointly selected qualified respondents in accordance with the following inclusion criteria: working as a MIC helper for no less than a month and still on the job; working in the hospital’s obstetric ward; and voluntary participation in the study.

A questionnaire link including three questionnaires was generated through wjx.cn. Ten MIC helpers were pre-surveyed to ensure the rationality of questions and choices in the questionnaires. Then questionnaires were sent by the researcher through WeChat to the MIC helpers in the hospitals of different cities in Zhejiang Province. The study was approved by the Institutional Review Board of the Women’s Hospital, Zhejiang University School of Medicine (approval no. IRB-20220151-R) and informed consent for participation in the study was signed online by the respondents prior to the survey. The questionnaire was anonymous to ensure data security. All questions were set as compulsory. In case of omission, after clicking “submit”, the respondents would be directed to the missed question to finish the answer, so that the completeness of the questionnaire was ensured.

### Measures

Demographic data were obtained by collecting participants’ age, educational background, marital status, length of working, grade of hospital, working hours, resting hours and monthly income.

Psychological Contract was measured by using the Psychological Contract Scale made by Li Yuan [33] which includes 39 items and consists of two sub-scales. One is the scale of organizational obligations to employees (organizational obligations for short), which includes organizational normal obligations (5 items), organizational interpersonal obligations (9 items) and organizational developmental obligations (7 items). The other is the scale of employee obligations to organizations (employee obligations for short), which includes employee normal obligations (6 items), employee interpersonal obligations (5 items) and employee developmental obligations (7 items). (Additional file 1). It adopted a Likert Rating Scale ranging from 1 to 5 (strongly disagree to strongly agree) and the respondents scored each item according to their psychological contract fulfillment. The higher the score is, the higher the level of psychological contract fulfillment. A score of 1–2 means low fulfillment level, 2–4 medium, 4–5 high. The Cronbach’α of the scale is 0.92 and the Cronbach’α for the six dimensions ranges from 0.673 to 0.909.

There is no readily available occupational wellbeing questionnaire specifically designed for the study of the MIC helpers. Considering that they are medical care aides serving puerperae and infants at hospital, after discussion with experts, this study used Occupational Wellbeing Scale for Medical Staff compiled by Hu Dongmei [34]. The scale covers 24 items in 5 dimensions: social support, value/competence, working environment, economic income, physical and mental health. The MIC helpers are under direct management of the MIC agencies that cooperate with hospitals, in order to design a more reasonable questionnaire for the survey, the word “hospital” in the original items such as “you are satisfied with the hospital’s welfare policy” was replaced by “agency”. “You are satisfied with current doctor-patient relationship” was adjusted into “you are satisfied with the relationship with those who you serve”. (Additional file 1). Each item is scored according to Likert Rating Scale ranging from 1 to 5 (strongly disagree to strongly agree). The overall Cronbach’α of the scale is 0.939 and the Cronbach’α for the 5 dimensions ranges from 0.815 to 0.898.

### Analysis

The SPSS 20.0 statistical software was used to do data analysis. Statistical methods included mean, standard deviation (SD), one-way analysis of variance (ANOVA), Pearson’s correlation, and stepwise multiple linear regression. Significance was predetermined at *p* < 0.05.

## Results

There were 260 respondents. Table 1 shows the majority of respondents were aged between 46 and 50 years old (39.6%). Over half of them (58.8%) had junior high school education. Most of the respondents have been working as MIC helpers for over 36 months (33.5%), followed by 20.4% for 6 to 12 months. The daily working hours of most respondents (48.8%) was 24 h and most of them (65.0%) got only a day off per week. The majority of respondents (43.8%) earned RMB 6,001 to 8,000 ($883.3 to 1177.6) a month.

**Table 1:** The distribution and ANOVA analysis of demographic data on occupational wellbeing (*N* = 260)

Variables	N	%	Occupational wellbeing		
			Mean ± SD	*F*	*P*
Age (years)				1.187	0.317
≤ 35	40	15.4	3.92 ± 0.66		
36–40	26	10.0	4.12 ± 0.6		
41–45	57	21.9	3.89 ± 0.66		
46–50	103	39.6	4.04 ± 0.58		
≥ 51	34	13.1	4.09 ± 0.61		
Educational background				0.064	0.979
Elementary school and below	15	5.8	4.02 ± 0.75		
Junior high school	153	58.8	4.02 ± 0.61		
Senior high school	81	31.2	3.98 ± 0.64		
Undergraduate and above	11	4.2	3.98 ± 0.3		
Grade of hospital				1.29	0.20
Class 3	197	75.8	4.03 ± 0.59		
Class 2	63	24.2	3.92 ± 0.69		
Length of working				3.330	0.011
1–6 months	48	18.5	3.85 ± 0.68		
6–12 months	53	20.4	3.82 ± 0.6		
13–24 months	39	15.0	4.11 ± 0.56		
25–36 months	33	12.7	4.07 ± 0.56		
> 36 months	87	33.5	4.13 ± 0.6		
Daily working hours				2.366	0.071
≤ 8	12	4.6	4.11 ± 0.39		
9–12	102	39.2	4.10 ± 0.59		
13–23	19	7.3	3.84 ± 0.71		
24	127	48.8	3.79 ± 0.63		
Days of rest per week				1.418	0.228
1	169	65.0	4.05 ± 0.6		
2	29	11.2	3.97 ± 0.75		
≥ 3	25	9.6	4.09 ± 0.45		
No day off	37	14.2	3.79 ± 0.65		
Monthly income (RMB)				5.580	0.000
≤ 4000	5	1.9	4.32 ± 0.12		
4001–6000	44	16.9	3.66 ± 0.67		
6001–8000	114	43.8	3.97 ± 0.55		
8001–10,000	69	26.5	4.15 ± 0.65		
10,001–15,000	24	9.2	4.32 ± 0.52		
> 15,000	4	1.5	4.21 ± 0.06		

Scores of items in the Occupational Wellbeing Scale were displayed in Table 2. The mean score of occupational wellbeing was 4.01 with a standard deviation of 0.62. The dimension scoring the highest was social support with a mean score of 4.41 (SD 0.64) and the lowest was physical and mental health with a mean of 3.12 (SD 1.27).

**Table 2:** The score of occupational wellbeing (*N* = 260)

	Items no.	Median	Mean	SD
Physical and mental health	6	3.17	3.12	1.27
Value/competence	6	4.33	4.27	0.70
Social support	5	4.60	4.41	0.64
Economic income	3	4.00	3.95	1.01
Working environment	4	4.25	4.27	0.75
Total	24	4.09	4.01	0.62

Scores of items in the Psychological Contract Scale can be seen in Table 3. The average score of psychological contract was 4.38 (SD 0.59) out of a maximum score of 5, indicating an overall positive level of psychological contract among the MIC helpers. For sub-scales, employee obligations (mean 4.50, SD 0.56) scored higher than organizational obligations (mean 4.25, SD 0.71). In the sub-scale of organizational obligations, organizational interpersonal scored highest with a mean score of 4.30 (SD 0.70), and organizational normal scored lowest with a mean score of 4.20 (SD 0.78). In the sub-scale of employee obligations, employee interpersonal scored highest with a mean score of 4.53 (SD 0.57), and employee developmental scored lowest with a mean score of 4.46 (SD 0.59).

**Table 3:** The score of psychological contract (*N* = 260)

	Items no.	Median	Mean	SD
Organizational obligations	21	4.19	4.25	0.71
Organizational normal	5	4.20	4.20	0.78
Organizational interpersonal	9	4.33	4.30	0.70
Organizational developmental	7	4.14	4.23	0.73
Employee obligations	18	4.67	4.50	0.56
Employee normal	6	4.83	4.52	0.57
Employee interpersonal	5	4.60	4.53	0.57
Employee developmental	7	4.57	4.46	0.59
Total	39	4.42	4.38	0.59

Single-factor analysis showed that statistically significant differences in the occupational wellbeing of MIC helpers based on the length of working and monthly income, with participants who worked for more than three years and earned between 10,001–15,000 yuan or less than 4000 yuan a month having the highest occupational wellbeing (Table 1).

According to the Pearson correlation analysis, psychological contract weas positively correlated with MIC helpers’ occupational wellbeing (r = 0.784, *p* < 0.001). Among the dimensions of psychological contract scale, organizational interpersonal obligations showed the strongest correlation with occupational wellbeing (r = 0.794, *p* < 0.001).

Stepwise multiple regression analysis showed that psychological contract and monthly income could explain 62.5% of variation in MIC helpers’ occupational wellbeing (*p* < 0.001). Participants who perceived higher psychological contract had better occupational wellbeing. Additionally, psychological contract is the most important predictor of occupational wellbeing and accounts for 54.8% of the total 62.1% variance explained by the linear regression, as shown in Table 4.

**Table 4:** Stepwise multiple linear regression of occupational well-being (*N* = 260)

	*β*	R^2^	*R*^*2*^*change*	*F*	*p* value
Step 1		0.080	0.073	11.200	0.000
Constant	3.384				0.000
Length of working	0.054				0.033
Monthly income	0.136				0.001
Step 2		0.625	0.621	142.167	< 0.001
Constant	0.300				0.102
Length of working	0.017				0.306
Monthly income	0.054				0.038
Psychological contract	0.794				0.000

## Discussion

### Occupational wellbeing

Reported level of overall occupational wellbeing in the MIC helpers was above average, higher than that of nurses [18, 35], indicating that they were relatively satisfied with the working condition. One of the highest scoring attributes in the Occupational Wellbeing Scale was ‘value/competence’, meaning they had a relatively high sense of professional value and professional identity, which has a positive impact on employees' loyalty, motivation, and sense of achievement [36]. Notably, the dimension of ‘physical and mental health’ averaged the lowest, but varied widely. In this survey, 4.6% of the MIC helpers work no more than 8 h per day, 20.8% of them have 2–3 days of rest a week, but most MIC helpers work long hours and 48.8% of them bonded with specific maternal and infants 24 h a day. This may account for the wide variation in MIC helpers' perceptions of "physical and mental health". The average labor intensity of the MIC helpers is greater than that of medical institutions, which is also an important reason behind the weak willingness of nursing graduates to serve as MIC helpers [37]. What’s more, fatigue caused by continuous long working hours will lead to an increase in the probability of mother–infant adverse events [38]. Therefore, it is both necessary and important for the MIC agencies to actively provide occupational health service for the MIC helpers, set reasonable working hours, and improve shift systems.

### Psychological contract

As an important part of people-oriented management, psychological contract is not only a mental bond between the MIC helpers and their administrators, but also a key element affecting their behaviors and attitudes [39]. This study revealed above average levels of MIC helpers’ psychological contract, higher than medical care helpers in hospitals in Taiyuan (mean 4.11, SD 0.63) [16] and care helpers in elderly care institution in Sichuan (mean 4.11, SD 0.46) [29] and Chongqing (mean 4.18, SD 0.35) [30]. This may be attributed to the working environment that allows MIC helpers to feel the vitality from young moms and newborns.

The findings of this study make clear that the higher the level of psychological contract, the higher MIC helpers' occupational wellbeing. Previous studies also showed that psychological contract was a protective factor for employers' occupational wellbeing, job satisfaction, wellbeing and organizational commitment, potentially reducing the intention to quit [24, 25]. These give administrators practical suggestions to improve outcomes for MIC helpers.

Looking at the difference of the scores of employee obligation and organizational obligation subscales, the MIC helpers subjectively believed what they contributed to the organization overweighed what they received from it, which is consistent with the research findings of Rao Yunshuang [30]. The mismatch between expectations and reality can result in disappointment and complaint or even a tendency to quit the job [28, 40]. In view of the lowest average score of organizational normal obligations, it indicated that the MIC helpers had higher expectations on income, welfare, and profession guarantee. This study found out 70.3% of the MIC helpers had a monthly income ranging from RMB6001 to 10,000 ($883.3 to 1472), better than the average annual salary of private sector employees in Zhejiang Province which is RMB60,521($8908.7) [41]. The MIC helpers have higher expectations of their incomes may be due to the longer working hours, which is the so-called more pay for more work” [42]. This gives administrators of mother–infant service agencies a potentially significant policy lever. On the one hand, administrators of agencies should improve the incentive mechanism for MIC helpers. The combination of tangible rewards such as monetary incentives and intangible rewards such as praise can enhance confidence in their individual skills and capabilities, which in turn had a direct impact on their work motivation [43]. On the other hand, MIC helpers’ expectations may need to be realigned. Increasing transparency from mother–infant service agencies regarding employees’ obligations and benefits may help this [25].

The current study revealed that the organizational interpersonal obligation is of utmost importance to MIC helpers' occupational wellbeing. Good interpersonal relationship in an organization is not only conducive to mother–infant care, but also surrounds the care helpers with a harmonious working atmosphere, in which the awareness of teamwork and cooperation is promoted among employees [44]. Support from different aspects is required to build up such atmosphere. The MIC helpers should equip themselves not only with caring skills but also communication skills with clients [30]. Registered nurses in obstetrics should regard them as partners and establish a good partnership with them. Last but not least, care and support from the organization’s administrators, and open communication with each other will enhance the sense of belonging and trust, thus increasing the probability of MIC helpers’ occupational wellbeing [24, 45].

When we specifically look at the MIC helpers’ perceptions of employees’ obligations, developmental obligations got the lowest score. It indicates the MIC helpers don’t have enough motivation and passion in improving their service level and long-term development of the organization. At present, most of the MIC helpers are over 41 years old with an education background of junior high school or below, hence they had poor learning ability and opportunities. Besides, there’s no clear promotion mechanism for this occupation [46]. Therefore, the administrators are supposed to provide them with necessary learning opportunities and acknowledge their skills so as to stimulate their confidence and improve their activity. In addition, administrators should set up reasonable, well-planned, and transparent promotion systems for the MIC helpers [47], so that they will have a clear understanding of their career development prospect and combine their personal development goals with the organizational development goals, which is conducive to increasing their enthusiasm for work, stabilizing the team of the MIC helpers in obstetrics and improving the service quality.

### Limitations

There are some limitations in this study. First of all, this study only surveyed the MIC helpers in obstetrics in Zhejiang Province, no other regions in China were covered. Secondly, the psychological contract scale used in this article was not designated for the target group of MIC helpers. And based on the scale for medical staff occupational wellbeing with some simple adjustments, the occupational wellbeing scale used here hasn’t gone through test of validity and reliability among the target group. Therefore, it is possible that some information unique to MIC helpers has been missed. Finally, this study just focuses on the perspective of MIC helpers and their understanding of organization obligations and employee obligations in psychological contract. It is suggested that future studies analyze the psychological contract from the viewpoints of both administrators and the MIC helpers, and compare the differences between them.

## Conclusion

To my knowledge, this article is the first study in China to research the psychological contract and occupational wellbeing of MIC helpers in obstetrics. The study found that their psychological contract was above average, and revealed that psychological contract and monthly income were significant predictors of occupational wellbeing, which explained 62.5% of the total amount of variance in MIC helpers’ occupational wellbeing. Future research and policy initiatives can build on these findings to explore the effective psychological contract management mechanism, and improve occupational wellbeing for the MIC helpers, so as to guide the MIC industry towards sustainable development.

## Supplementary Information

**Additional file 1.** Psychological Contract Scale and Occupational Wellbeing Scale.

## Data Availability

The datasets used and/or analyzed during the current study are available from the corresponding author on reasonable request.

## Abbreviations

MIC: Mother–infant care
SD: Standard deviation
