# Supplementary material for: The relationship between psychological contract and occupational wellbeing of mother–infant care helpers in Zhejiang Province
Source: Hum Resour Health. 2023 Mar 1;21:15. doi: 10.1186/s12960-023-00793-w (PMC9976691; doi:10.1186/s12960-023-00793-w)
Supplement: Supplementary file 1 — Additional file 1. Psychological Contract Scale and Occupational Wellbeing Scale. [file 12960_2023_793_MOESM1_ESM.docx]

**1.心理契约量表**

**Psychological contract scale**

**1.1 Organizational obligations to employees**

|  | 项目Items | 非常不符合  Not at all | 比较不符合  Slight extent | 不确定  Moderate extent | 比较符合  Great extent | 非常符合  Very great extent |
| --- | --- | --- | --- | --- | --- | --- |
| 1 | 所在单位给我提供了稳定的工作保障  My work unit provides me with stable job security | 1 | 2 | 3 | 4 | 5 |
| 2 | 所在单位根据我的工作业绩发放工资和奖金  My work unit pays me salary and bonus according to my performance |  |  |  |  |  |
| 3 | 所在单位关怀我的个人成长和生活  My work unit cares about my personal growth and life |  |  |  |  |  |
| 4 | 所在单位给我提供的工作富于挑战性  The work provided by my unit is challenging |  |  |  |  |  |
| 5 | 所在单位给我提供了事业发展的机会  My work unit offers me a chance for career development |  |  |  |  |  |
| 6 | 所在单位给我提供了工作的自主权  My work unit provides me with autonomy in my work |  |  |  |  |  |
| 7 | 这个单位的上下级关系和谐、友好  The relationship between superiors and subordinates in this unit is harmonious and friendly |  |  |  |  |  |
| 8 | 这个单位中同事之间相互信任和帮助  Colleagues in this unit trust and help each other |  |  |  |  |  |
| 9 | 这个单位给我提供了友善而融洽的工作环境  This unit provides me with a friendly and harmonious working environment |  |  |  |  |  |
| 10 | 这个单位给我提供了学习和培训机会  This unit provides me with learning and training opportunities |  |  |  |  |  |
| 11 | 与其他单位相比，这个单位给我提供的待遇比较公平合理  Compared with other units, the treatment provided by this unit is fairer and more reasonable |  |  |  |  |  |
| 12 | 这个单位给我提供不错的福利待遇  This unit provides me with a good welfare package |  |  |  |  |  |
| 13 | 这个单位十分重视自己的员工  The unit attaches great importance to its employees |  |  |  |  |  |
| 14 | 这个单位提供了合作的工作氛围  The unit offers a collaborative work atmosphere |  |  |  |  |  |
| 15 | 这个单位给我提供晋升的空间  This unit offers me room for advancement |  |  |  |  |  |
| 16 | 这个单位能真诚地对待自己的员工  The organization treats its employees in good faith |  |  |  |  |  |
| 17 | 这个单位在做重大决策之前，会充分考虑员工的意见  The unit will give full consideration to the opinions of its employees before making major decisions |  |  |  |  |  |
| 18 | 这个单位能让我发挥技术和专长，学有所用  This unit allows me to give full play to my skills and expertise |  |  |  |  |  |
| 19 | 在这个单位给我提供了资源充分的工作环境  This unit provides me with a well-resourced working environment |  |  |  |  |  |
| 20 | 在这个单位中我能得到良好的有关工作方面的指导  I can get good guidance about my work in this unit |  |  |  |  |  |
| 21 | 这个单位经常肯定我的贡献的成绩  The unit often recognizes my contribution to the grades |  |  |  |  |  |

**1.2 Employee obligations to organizations**

|  | 项目Items | 非常不符合  Not at all | 比较不符合  Slight extent | 不确定  Moderate extent | 比较符合  Great extent | 非常符合  Very great extent |
| --- | --- | --- | --- | --- | --- | --- |
| 1 | 在工作需要时，可以加班加点完成工作  Work overtime to complete the work as required | 1 | 2 | 3 | 4 | 5 |
| 2 | 忠诚于本单位  Be loyal to your unit |  |  |  |  |  |
| 3 | 希望本单位发展比其他单位更好  I hope this unit will develop better than other units |  |  |  |  |  |
| 4 | 自觉帮助单位做额外的工作而不计较有无报酬  Volunteer to help the organization do extra work without worrying about pay |  |  |  |  |  |
| 5 | 如果辞职会提前向单位打招呼  If I resign, I will explain the situation to the employer in advance |  |  |  |  |  |
| 6 | 保守单位的一切秘密  Keep all the secrets of the unit |  |  |  |  |  |
| 7 | 与周围同事保持良好的合作关系  Maintain good cooperative relationship with colleagues around |  |  |  |  |  |
| 8 | 在工作团队中起带头作用  Take the lead in the work team |  |  |  |  |  |
| 9 | 为周围的同事提供额外的帮助  Offer extra help to coworkers |  |  |  |  |  |
| 10 | 为单位的利益而做出个人牺牲  Making personal sacrifices for the good of the organization |  |  |  |  |  |
| 11 | 在外人面前宣传和维护单位的形象  Promote and maintain the organization's image to outsiders |  |  |  |  |  |
| 12 | 只要单位需要，我会接受工作变动  As long as the organization needs, I will accept job changes |  |  |  |  |  |
| 13 | 积极为单位的发展献计献策  Actively contribute to the development of the unit |  |  |  |  |  |
| 14 | 不断学习母婴护理技能，提高自己的业务水平  Continue to learn maternal and infant nursing skills, improve their professional level |  |  |  |  |  |
| 15 | 在工作团队中共享信息，一同解决问题  Share information and solve problems together in a work team |  |  |  |  |  |
| 16 | 自觉提高自己的业务水平，以适应单位发展的需求  Consciously improve their business level, to meet the needs of the development of the unit |  |  |  |  |  |
| 17 | 对单位的发展投入全身心的热情  Full enthusiasm for the development of the unit |  |  |  |  |  |
| 18 | 配合上级领导完成工作安排  Cooperate with superior to complete the work arrangement |  |  |  |  |  |

Note:

1. The subscale of organizational obligations to employees includes organizational normal obligations (1,2,11,12,19), organizational interpersonal obligations (3,7,8,9,13,14,16,20,21) and organizational developmental obligations (4,5,6,10,15,17,18). The subscale of employee obligations to organizations includes employee normal obligations (1,2,3,5,6,12), employee interpersonal obligations (7,8,9,15,18) and employee developmental obligations (4,10,11,13,14,16,17).
2. The confirmatory factor analysis demonstrated a good model fit to the three-factor 'organizational obligations' model: X^2^/df=2.832, RMSEA =0.100, SRMR =0.071, CFI =0.83, IFI=0.83; The three-factor 'employee obligations' model also fit well: X^2^/df=3.570, RMSEA =0.119, SRMR =0.074, CFI =0.79, IFI=0.79. Cronbach's alpha of 0.92 for the total score and 0.673-0.909 for the item scores demonstrated high internal consistency.

**2. 职业幸福感量表 Occupational Wellbeing Scale**

|  | 项目Items | 非常不符合  Not at all | 比较不符合  Slight extent | 不确定  Moderate extent | 比较符合  Great extent | 非常符合  Very great extent |
| --- | --- | --- | --- | --- | --- | --- |
| 1 | 您身体状况经常处于亚健康状态  Your physical condition is often in sub-health state | 1 | 2 | 3 | 4 | 5 |
| 2 | 工作给您的身体健康带来了负面的影响  Work is taking a toll on your health |  |  |  |  |  |
| 3 | 目前您的工作压力大  You are under a lot of pressure at work |  |  |  |  |  |
| 4 | 工作让您心力交瘁，甚至想现在就辞职  Your job is burning you out and you want to quit right now |  |  |  |  |  |
| 5 | 在工作中您时常感到压抑,情绪低落,脾气变坏有时很难控制  At work, you often feel depressed, depressed, and have a bad temper that is sometimes difficult to control |  |  |  |  |  |
| 6 | 您感觉工作很累，没有时间和精力做您喜欢的事  You feel tired at work and don't have the time or energy to do what you like |  |  |  |  |  |
| 7 | 您觉得您的职业是神圣的  You feel that your profession is sacred |  |  |  |  |  |
| 8 | 您对现在的工作状态满意  You are satisfied with your current working status |  |  |  |  |  |
| 9 | 您对自己的社会地位满意  You are satisfied with your social status |  |  |  |  |  |
| 10 | 您喜欢目前的工作  You like your current job |  |  |  |  |  |
| 11 | 您对自己从事的工作有成就感  You have a sense of accomplishment in your work |  |  |  |  |  |
| 12 | 您认为您目前工作的晋升机会较多，有良好的发展空间  You think your current job has more opportunities for advancement and good room for development |  |  |  |  |  |
| 13 | 您认为单位有和谐的人际关系  You think the organization has harmonious interpersonal relationship |  |  |  |  |  |
| 14 | 家人和朋友支持您的工作  Family and friends support you in your work |  |  |  |  |  |
| 15 | 领导能够支持您的工作  Leaders can support your work |  |  |  |  |  |
| 16 | 同事能够支持您的工作  Colleagues can support your work |  |  |  |  |  |
| 17 | 您在工作中遇到问题时，能够从上级那里获得帮助  When you have a problem at work, you can get help from your superiors |  |  |  |  |  |
| 18 | 您对您现在的收入水平满意  You are satisfied with your current level of income |  |  |  |  |  |
| 19 | 您认为目前自己在工作上的付出与 收入成正比  You think that what you are currently doing at work is proportional to your income |  |  |  |  |  |
| 20 | 您对目前单位的福利制度满意  You are satisfied with the welfare system of your current agency |  |  |  |  |  |
| 21 | 您对目前的医患关系满意  you are satisfied with the relationship with those who you serve |  |  |  |  |  |
| 22 | 您对医院所提供的工作环境感到满意  You are satisfied with the working environment provided by the agency |  |  |  |  |  |
| 23 | 您对单位的管理制度、晋级制度满意  You are satisfied with the management system and promotion system of the unit |  |  |  |  |  |
| 24 | 单位能够为您提供较好的进修机会  The unit can provide you with better opportunities for further study |  |  |  |  |  |

Note:

The scale covers 5 dimensions: physical and mental health (1-6 items), value/competence (7-12 items), social support (13-17 items), economic income (18-20 items), working environment (21-24 items)
